# Claude Bernard, the Founder of Modern Medicine

**DOI:** 10.3390/cells11101702

**Published:** 2022-05-20

**Authors:** René Habert

**Affiliations:** Laboratory of Development of the Gonads, UMR-008 Genetic Stability Stem Cells and Radiations, Université de Paris, 92265 Fontenay-aux-Roses, France; r.habert@orange.fr

Claude Bernard is the first and one of the very few French scientists to have been honored with a national funeral. This shows how much this doctor–researcher left an essential mark on the history of science and medicine (Figure 1).

## 1. From Beaujolais to Paris

Claude Bernard was born in 1813 in Saint Julien (69640). It is a small village in the heart of Beaujolais, 30 km north of Lyon. 

**Figure 2:**
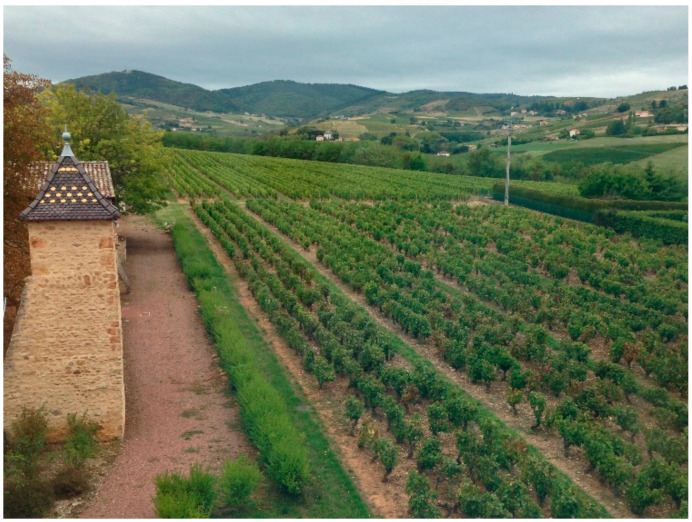
Claude Bernard’s family’s pigeon house and vines in Saint Julien in Beaujolais.

It has been written too often that Claude Bernard came from a modest family of wine growers. This is obviously wrong, since the son of a modest winegrower was not entitled to learn to read and write before education for all was promoted by Paul Bert, a pupil of Claude Bernard, and by Jules Ferry. Therefore, Claude Bernard would never have become a doctor if his parents had been modest wine growers. His family was a family of the rural bourgeoisie. His father was a wine merchant and landowner, and his mother had brought the family home and adjoining hectares of vines as a dowry (Figure 2). After rather poor studies, Claude Bernard failed the Baccalaureate in 1831. Then, he became an apprentice in pharmacy near Lyon, but preferred to devote himself to writing plays. Fortunately for science, his plays were not successful. At the age of 20, he picked up the wrong number during the draw to perform (or not) military service, which lasted 7 years at the time. His family paid for a substitute who had drawn a good number, and Claude Bernard could thus go to Paris to resume his studies, pass his Baccalaureate, and then move towards medical studies.

He performed his medical training education in various departments, but it was through contact with François Magendie, holder of the Chair of Medicine at the Collège de France, that he found his vocation and became passionate about research. He became Magendie’s assistant in 1841, and his mentor gradually saw him as his successor. His first discovery, in 1844, experimentally demonstrated the role of one nerve, the tympanic cord, in the transmission of the sensation of taste from the tongue to the brain.

He was a tireless worker. The reading of his experiment notebooks, all kept at the Collège de France, induces vertigo due to the number of experiments and ideas that he records in one day.

In 1851, he had already received three prizes from the Academy of Sciences. In 1855, he succeeded Magendie. At the same time, he was a professor at the Sorbonne and then at the National Museum of Natural History. He was elected to three academies in France (the Academy of Sciences in 1854, the Academy of Medicine in 1861, and the French Academy in 1868) and to several academies abroad. Today, it is still the statue of Claude Bernard that welcomes us at the entrance of the Collège de France in Paris.

**Figure 3:**
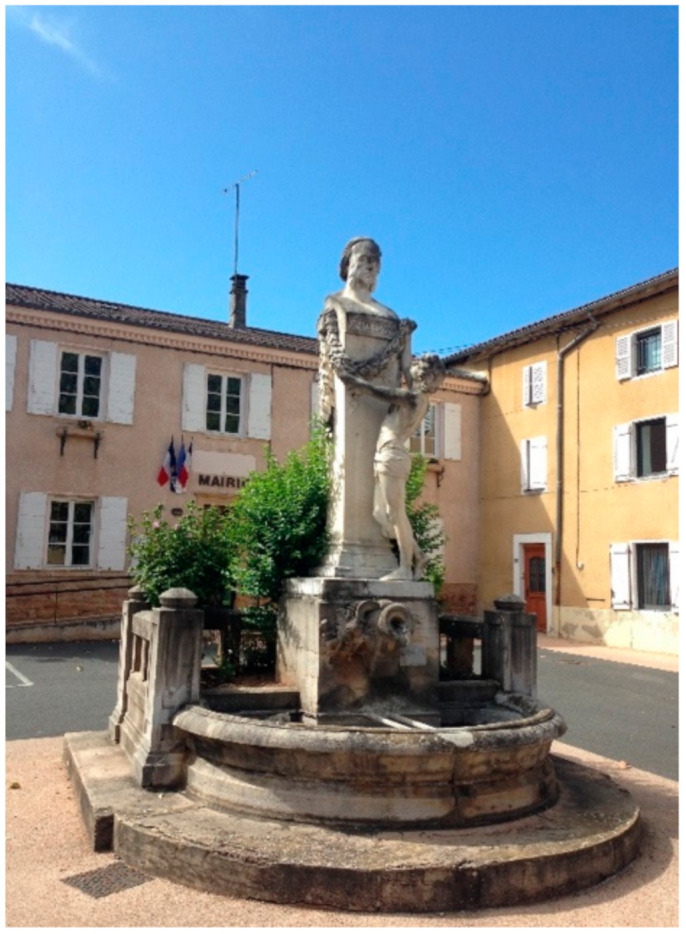
Statue of Claude Bernard on the village square in Saint Julien in Beaujolais—69640—work by Arthur de Gravillon. Decided by the municipal council of Saint Julien as soon as 1878, and inaugurated in 1885, this marble statue was the first one erected in honor of Claude Bernard.

C. Bernard remained attached to Saint Julien in Beaujolais, his native village, all throughout his life. In 1861, he acquired the beautiful mansion next to his birthplace, which is now the Claude Bernard Museum. He returned regularly to Saint Julien, especially during the grape harvest, taking advantage of the brand new Paris–Lyon railway line that opened in July 1854 (Figure 3).

## 2. Claude Bernard Revolutionized Biology and Medicine

To simply present the immense work and genius of Claude Bernard, his discoveries can be classified into three levels.

### 2.1. Level One: Acquisition of New Knowledge

His discoveries are as numerous as they are varied. Moreover, they are of the highest importance: the role of the stomach, bile, and the pancreas in digestion, the nervous regulation of numerous functions (digestion, circulation, motricity, etc.), the mechanisms of fermentation, the study of anesthetics, etc. His best-known discovery is the glycogenic function of the liver. When glucose is in excess in the blood, the liver captures it and stores it as glycogen, a molecule discovered by C. Bernard. Conversely, when the blood is too low in glucose, the liver transforms its glycogen reserves into glucose, which it pours into the blood. 

### 2.2. Level Two: Development of New Concepts

The concepts designed by Claude Bernard became the foundations of modern physiology and medicine. 

**Figure 4:**
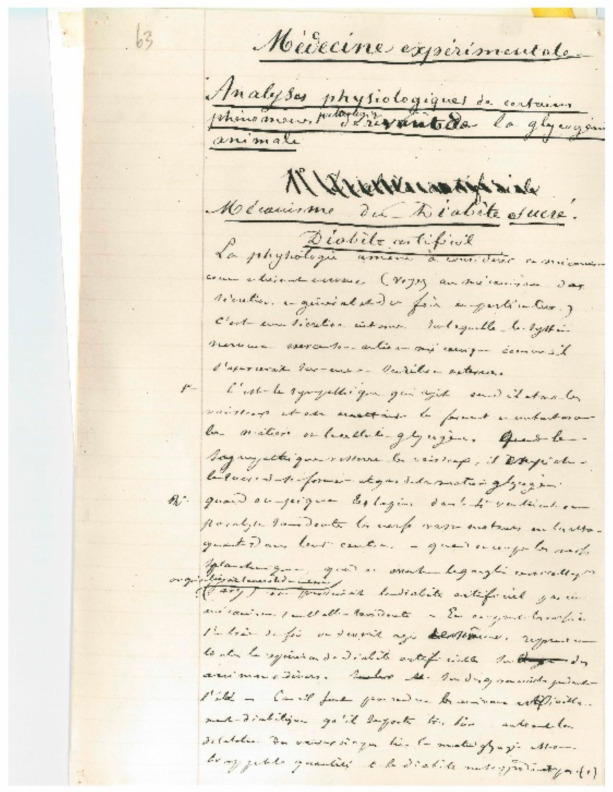
Claude Bernard’s manuscript of a lesson in experimental medicine. Musée Claude Bernard, Communauté d’Agglomération Villefranche Beaujolais Saône. France.

He discovered that biochemical reactions can be reversible (glucose–glycogen). Before Claude Bernard, it was known that some organs are able to produce excretions outside the organism (urine, bile, sweat, tears, etc.). By showing that the liver secretes glucose into the blood, Claude Bernard showed that organs can also produce and secrete molecules into the blood, and he conceptualized the notion of internal secretion. Before Claude Bernard, it was thought that each organ had only one function. By showing that the liver not only produced bile poured into the digestive tract but also secreted glucose into the blood, Claude Bernard showed that the same organ can perform several functions. Claude Bernard also developed the idea that the living world shared common properties, since both animals and plants can synthetize carbohydrates such as glycogen and starch, respectively. It was a revolutionary idea of the uniqueness of the living world (Figure 4).

His main contribution to physiology and medicine is probably the creation of the concept of «*milieu intérieur*» and its constancy, which was later called homeostasis. Claude Bernard wrote “*For the animal there are really two environments: an external environment in which the organism is placed, and an internal environment in which the elements of the tissue live…. The fixity of the internal environment is the condition of free and independent life*” [1]. This concept is such an important one that the term «*milieu intérieur*» is still often kept in French in English language publications. 

He also led a fierce struggle to deny vitalism. Vitalism was a dominant theory at the time of Claude Bernard. According to this theory, life cannot be explained solely by the laws of physics and chemistry: life was also governed by an unidentified «vital principle» that stands above these laws. As an example, in 1877, at the end of his life, Claude Bernard discovered that alcoholic fermentation was the result of the action of molecules then called “soluble ferments”, and today called “enzymes”. Claude Bernard’s results were published by Marcellin Berthelot after his death. However, this was virulently contradicted by Louis Pasteur, who was convinced that whole, living yeast was necessary for fermentation, and the controversy lasted for years. It was not until 1897, two years after Pasteur’s death, that the German chemist Eduard Büchner showed that yeast acts by secreting enzymes, putting an end to the vitalist theory of fermentation supported by Pasteur.

Finally, the new concepts developed by Claude Bernard were often in opposition to the ideas and theories of the time. He wrote that “*It is what we think we already know that often prevents us from learning*” [2] and “*Theories can only be destroyed by new theories*” [2]. 

### 2.3. Level Three: Creation of a New Scientific Approach: The Experimental Medicine

At the time of Claude Bernard, medicine described and classified illnesses. Treatments were empirical. Claude Bernard revolutionized medicine and medical research by conceptualizing a method he called “*experimental medicine*”, which still forms the basis of countless medical advances today. 

**Figure 5:**
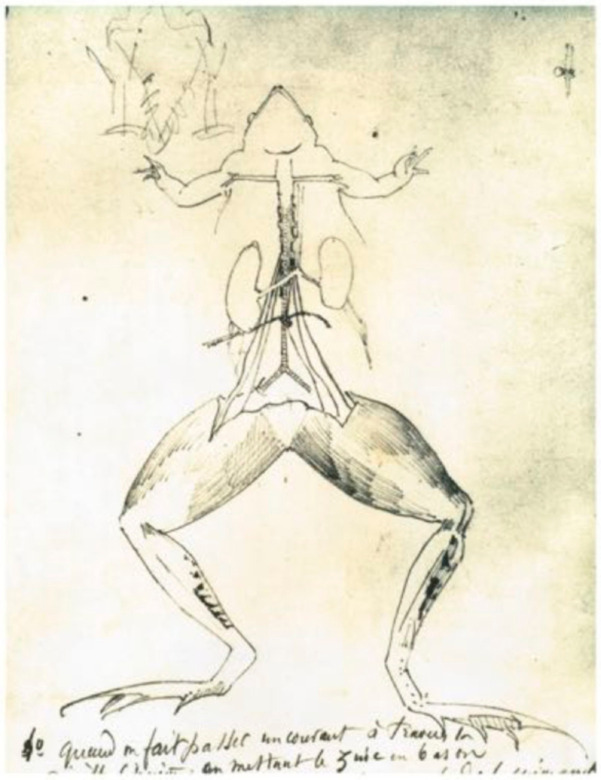
A dissection scheme drawn by Claude Bernard. Musée Claude Bernard, Communauté d’Agglomération Villefranche Beaujolais Saône. France.

His reasoning is based on four pillars that logically follow one another:

1. A disease is only a disruption of the normal functioning of the body.
2. To treat a patient, it is therefore necessary to understand this normal functioning.
3. To understand this normal functioning, it is necessary to conduct animal experiments (Figure 5).
4. To carry out these experiments, a hypothetico-deductive approach must be applied.

Claude Bernard described this approach in his book *Introduction à l’étude de la médecine expérimentale* (1865). He sums it up as follows:

“The complete scientist is one who embraces both theory and experimental practice”.

1. He states a fact.
2. In regard to this fact, an idea arises in his mind.
3. In view of this idea, he reasons, institutes an experience, and imagines as well as realizes its material conditions.
4. From this experience result new phenomena which must be observed, and so on.

At the end of the 20th century, pedagogues gave the acronym OPHERIC to this hypothetico-deductive approach: first, we make an Observation which poses a Problem, that is to say, a question. Then, we make a Hypothesis to solve this problem. Then, we design and carry out an Experiment to invalidate or confirm this hypothesis. The Results of this experiment are noted. Finally, we Interpret these results and draw a Conclusion. Of course, a discovery always opens new questions; this is how medical science always progresses today.

Claude Bernard summed up this symbiosis between thought and experimentation: “*A skilled hand, without the head to direct it, is a blind instrument; the head without the realizing hand remains powerless*” [2].

In conclusion, Claude Bernard is clearly the founder of modern physiology and medicine. The chemist Jean-Baptiste Dumas said of him “*He is not a great physiologist; he is the physiology itself*” [3]. Thus, in the middle of the 19th century, one man alone could create a new science.

## 3. So, Why Is Claude Bernard This Great Misunderstood?

Besides the fact that Claude Bernard’s personality was more oriented towards hard work in the laboratory than towards careerist approaches, we can identify several reasons for the fact that he is poorly recognized:

(1) His findings are in a specialized field. The very term “physiology” is a word little understood by the general public. Unfortunately, very few people can understand the discoveries made by Claude Bernard.
(2) Claude Bernard did not develop a therapy, unlike Pasteur, whose discovery of microbes immediately led to the development of vaccines. It was not until the 20th century that the molecules, and in particular the hormones, involved in the mechanisms discovered by C. Bernard were identified, thus allowing the creation of drugs. This is particularly evident in the fields of neurology and nutritional physiology (diabetes, obesity), the foundations of which were laid by Claude Bernard.
(3) His thought was so correct that it imposed itself as obvious, and everyone has forgotten that it was Claude Bernard who was the author. Everyone knows that our blood tests must be normal to be healthy, but too few people know that it was Claude Bernard who first discovered that blood has a constant composition. When a patient consults a doctor, and when his first question is not to ask what his treatment will be, but what is the cause of his pathology, he reproduces Bernardian thought without knowing it. When a researcher develops an experimental protocol based on an explanatory hypothesis, he steps on the shoulders of Claude Bernard without knowing it.

A new association has just been created to make Claude Bernard better known: The Association Claude Bernard (ACB). Its objectives are:

- To promote the figure and work of Claude Bernard.
- To support the Claude Bernard Museum in Saint Julien in Beaujolais.
- To promote scientific culture among a wide audience, in particular among young people, and thus arouse a passion for the knowledge of life.

**Figure 6:**
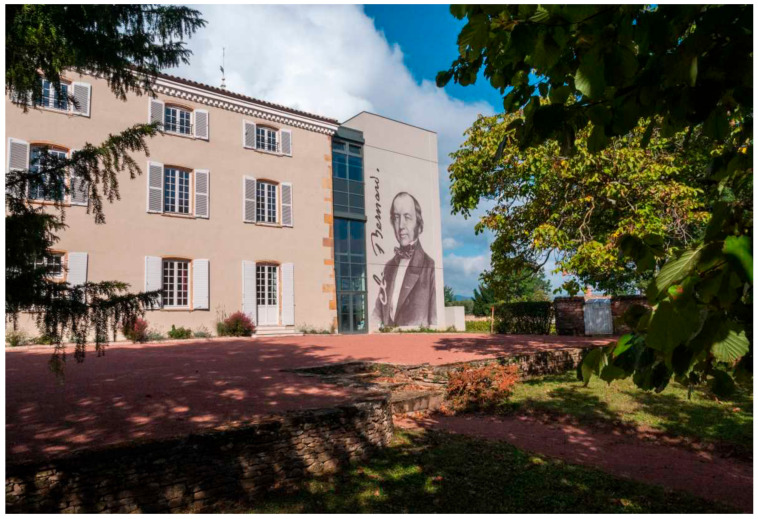
The Claude Bernard Museum in Saint Julien in Beaujolais.

Recently, the ACB created a video explaining the concept of homeostasis as simply as possible [4]. You can consult the website of the Association Claude Bernard [5], and you can also support this Association. You can also visit the Claude Bernard Museum in Saint-Julien in Beaujolais, 69640, France (Figure 6).

## Figures and Tables

**Figure 1:**
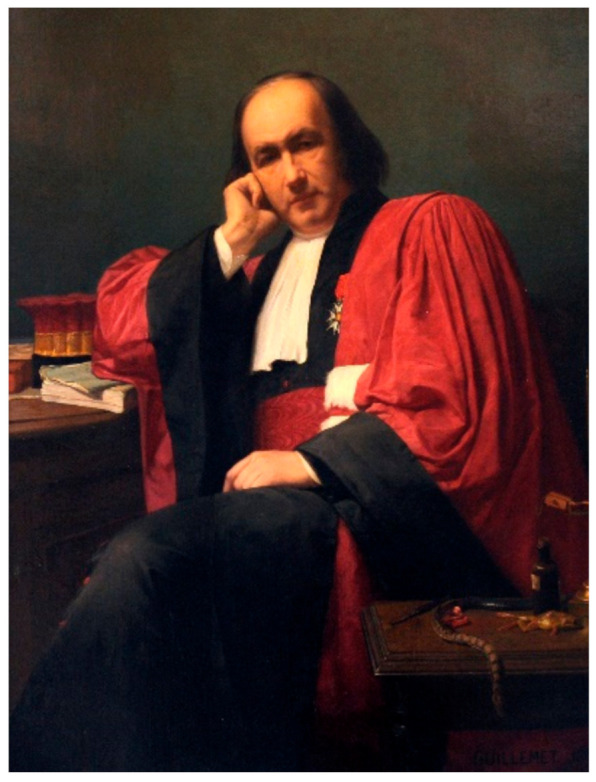
Claude Bernard in 1861 in his dress of a professor of the Faculty of Sciences. Unknown painter. Musée Claude Bernard, Communauté d’Agglomération Villefranche Beaujolais Saône. 69640, FRANCE.
